# Rational Design of Photothermal and Anti-Bacterial Foam With Macroporous Structure for Efficient Desalination of Water

**DOI:** 10.3389/fchem.2022.912489

**Published:** 2022-05-11

**Authors:** Zhifen Wang, Jin Niu, Juanxia Wang, Yucang Zhang, Guoqiang Wu, Xiaoyun Liu, Qun Liu

**Affiliations:** ^1^ College of Materials Science and Engineering, Hainan University, Haikou, China; ^2^ College of Ocean Food and Biological Engineering, Jimei University, Xiamen, China

**Keywords:** liquified chitin, silver nanoparticles, photothermal materials, solar distillation, anti-bacterial

## Abstract

With the environmental deterioration and the rise in demand for sustainability, the lack of freshwater resources has emerged as a global concern. To address this issue, the desalination of water using solar evaporation is centered on as a promising approach. In this study, we designed a light and photothermal liquefied-chitin-based polyurethane foam to achieve efficient water evaporation benefiting from their powerful solar spectral absorption, low thermal conductivity, quick transportation of water, hierarchically porous structures, and anti-biofouling natures. Moreover, because of the introduction of nano-silver, the newly developed foam exhibits considerable antibacterial ability and improved photothermal performance. Notably, the low thermal conductivity of the foam can reduce the loss of absorbed solar heat, whereas its large porous structure provides a smooth water transport channel. More importantly, with the assistance of heat, polyacrylamide hydrogels adhering along with the pores rapidly absorb and desorb water molecules, promoting the evaporation of water and improving solar energy conversion efficiency. Ultimately, under irradiation by one sunlight, the proposed material demonstrated a water evaporation rate and solar photothermal conversion efficiency of 2.44 kg m^−2^ h^−1^ and 153.2%, respectively.

## Introduction

In addition to population growth and exacerbated environmental pollution, the scarcity of freshwater resources has become a severe global concern (Ahmed et al., 2019). Inspired by the ever-important hydrological cycle, which replenishes terrestrial freshwater supplies using natural evaporation, solar evaporative seawater desalination is appearing to be a promising solution (Dalvi et al., 2015; Zhu et al., 2018; Xu et al., 2019b). In a solar evaporative seawater desalination system, heat is generated through interactions between incident sunlight and photothermal materials. After absorption by photothermal conversion materials, the incident solar energy is converted into heat energy, which is then used to heat seawater to create steam. Based on the position of the photothermal conversion material in the water body, solar evaporative seawater desalination systems can be classified into three categories: bottom heating conversion systems, volume heating conversion systems, and interface heating conversion systems (Tao et al., 2018). Bottom heating conversion systems and volume heating conversion systems have low evaporation efficiencies because of high heat losses and low surface temperatures. On the other hand, interface solar heating systems operate on the principle of floating a two-dimensional or three-dimensional photothermal conversion material on the water interface, to generate steam, in which the photothermal conversion material surface is selectively heated rather than the entire water body. The main body water has a temperature similar to that of the ambient environment, which minimizes heat losses due to migration from the water to the environment and eliminates heat losses from the bottom water body in the steam generation, thus achieving higher solar steam generation efficiencies.

In terms of the materials known for photothermal conversion, metal-based nanomaterials, nowadays, are considerable since they carry numerous movable electrons for heat absorption. Under the irradiation of light, free electrons on the metal surface oscillate with the incident light, resulting in a surface plasmon resonance effect (SPR). The strong plasmon resonance of the metal particles creates hot electrons when exposed to incident light with a matching oscillation frequency, which has been the subject of a number of studies. For example, Orrit was the first to report the occurrence of a plasmona-photothermal effect in gold nanoparticles (Boyer et al., 2002). Thereafter, Halas et al. investigated the use of gold nanoparticles in solar water vapor (Fang et al., 2013; Neumann et al., 2013), and Deng et al. fabricated gold nanoparticle films that were able to float automatically in air and further prepared free-standing photothermal films by depositing gold nanoparticles on air-loaded paper (Wang et al., 2014). During the usage of these self-floating photothermal films, of which the localized heating only occurrence at the air-water interface contributes to the increase of water evaporation efficiency (Liu et al., 2015; Yu et al., 2015; Liu et al., 2016). Besides, in view of the inherently narrow absorption bandwidth of well-sorted gold nanoparticles, Kim et al. prepared ultra-broadband light-absorption, flexible thin-film black gold films via adiabatic plasmonic nanofocusing (Bae et al., 2015), finally obtaining a high solar absorption efficiency up to 91%. To further expand the absorption of solar radiation, Zhu et al. loaded gold nanoparticles with random dimensions and distributions into nanoporous templates, achieving 99% absorbance in the wavelength range 400 nm to 10 μm and enabling the water evaporation efficiency to reach 90% under 4-sun irradiation (Zhou et al., 2016). Concurrently, the adhesion and pollution of the microorganisms such as various bacterial, is another key point that needs to be dealt with since it directly affects the lifetime of photothermal materials for practical application (Yang et al., 2020; Zhang et al., 2022). Hence, to achieve the above target, large number of flexible photothermal devices by hybridizing metal-based materials and polymers have been designed (Gao et al., 2018; Xu et al., 2019a).

However, many approaches for solar desalination currently suffer from the issues of relatively low efficiencies, complex synthesis, high cost, and poor antimicrobial ability (Fan et al., 2022). By comparison, the major required preconditions were taken into consideration in this study. Based on five strategies: powerful solar spectral absorption, low thermal conductivity, quick transportation of water, hierarchically porous structures, and anti-biofouling, we ultimately fabricated polyurethane-based photothermal foam for efficient and rapid solar conversion. In the preparation process, a simple and universal strategy to disperse photothermal silver nanoparticle and liquified chitin in polymer matrix was used in foaming formation. Through the *in-situ* generation of a polyacrylamide water-absorbing material in the pores of polyurethane foam, a quick water-attracting channel was then manufactured since polyacrylamide can easily release water under external heat (less than 40°C). Furthermore, the low thermal conductivity of the foam can reduce heat energy losses from the water body. As a result, the temperature on material surface can quickly overtake 100°C, and the final solar photothermal conversion efficiency exceeded 90%, which is much higher than those of most photothermal materials reported in literature.

## Materials and Methods

### Experimental Part

In a three-necked glass flask equipped with a stirrer, condenser, and thermometer, chemicals were added in the order of mixed solvent of PEG 400 and glycerin (28 g, 4:1, w/w), sulfuric acid (2 g) and chitin (4 g). The mixture was stirred at 160°C for 90 min and then cooled down to room temperature (Zhang et al., 2018).

Preparation of nano-silver-based polyurethane material: To prepare the chitin-liquefied polyurethane foam, nano-silver is first put into a mixing vessel, after which polyurethane is added. The mixture is stirred evenly using an electric mixer, and then a certain amount of isocyanate is added. The resulting mixture is stirred at a high speed, and then quickly poured into a mold to allow foam molding at room temperature. The mixture is allowed to stand and cure for 1 h, and then demolded. The obtained material is a silver-based polyurethane foam, which is hereafter referred to as Ag, and the preparation process is shown in Supplementary Figure S1.

On the other hand, a certain amount of polyacrylamide (PAM) is dissolved and stirred in water, after which potassium peroxodisulfate, N,N′-methylenebisacrylamide, and tetramethylethylenediamine are added simultaneously. The obtained mixture is then stirred evenly. Subsequently, the above Ag/polyurethane foam is placed into the mixture to polymerize the acrylamide hydrogel in the foam, thereby producing a polyurethane syntactic foam, which is hereafter referred to as Ag/PAM.

### Performance and Structural Characterization

Infrared spectroscopy analysis: A scanning test was performed on the polyurethane foam in the range 4,000–450 cm^−1^ using a Spectrum One Fourier infrared spectrometer (Perkin-Elmer, United States); prior to testing, the samples were prepared via potassium bromide tableting.

Scanning electron microscope analysis: The morphologies of the samples were observed using a Phenom ProX scanning electron microscope (Phenom Scientific Instrument (Shanghai) Co., Ltd.); prior to observation, the samples were sprayed with gold.

Thermogravimetric analysis: The thermal stabilities and thermal decomposition behaviors of the samples were tested using a thermogravimetric analysis (TGA) / differential scanning calorimetry (DSC) synchronous thermal analyzer (METTLER-TOLEDO Instruments, Switzerland). Each sample weighed approximately 10 mg. In a nitrogen atmosphere, the temperature was increased from 30 to 600°C at a heating rate of 10°C/min, and the thermogravimetric (TG) curve was recorded.

Contact angle test: The hydrophilicity of the polyurethane foam was tested using a contact angle tester.

Thermal conductivity test: The thermal conductivity of the foam was measured using a thermal conductivity tester with a sample size of 20 cm × 20 cm × 5 cm.

Absorbance test: A Lambda 750s ultraviolet–visible–near-infrared (UV–Vis–NIR) spectrometer integrating sphere unit and reflectance automatic measuring unit were used to record the diffuse reflectance and transmittance of the samples. The measurement wavelength range was 250–2,500 nm.

Evaporation efficiency test: Each sample was placed in a beaker filled with water and allowed to float on the water surface. A solar simulator with an optical density of 1 kW m^−2^ was used to simulate a one-sun irradiation sample, and the 30-min water evaporation rate was measured under steady-state conditions. An electronic analytical balance was used to measure the weight change due to water evaporation with an accuracy of 0.1 mg. The surface temperatures of the samples were recorded using an infrared thermal imager.

DSC measurement and thermodynamic simulation of water evaporation enthalpy in each sample: The evaporation enthalpy of each sample in reclaimed water was measured using a METTLER TOLEDO DSC 822^e^ DSC. A 10-mg sample was placed in a DSC aluminum crucible, and a hole was punched in the crucible lid to allow water evaporation. Measurements were performed from 20 to 180°C at a heating rate of 5°C/min under a nitrogen flow of 50 ml/min (Guo et al., 2020).

Test of antibacterial properties of materials: The antibacterial properties of the carbon-based materials were determined via plate counting. For this purpose, standard *E. coli* (TCC 25922, Gram-negative bacteria) were cultured on nutrient agar subjected to dry heat sterilization for two generations. This culture was used as a strain for the antibacterial tests. The nutrient agar medium was first sterilized in an autoclave at 121°C and 101.3 kPa for 30 min, poured into a petri dish, and cooled to room temperature as a medium. Under aseptic conditions, a certain number of sterilized samples were taken and placed in 7-ml *Staphylococcus aureus* culture solution, which was cultured in a shaker at 37°C for 24 h. Subsequently, 50 µL was obtained from the bacterial culture solution and diluted 104 times, followed by dropwise addition of 50 µL to the nutrient agar. A coating rod was used to evenly disperse the bacterial solution on the nutrient agar. After 24 h of culturing, the number of viable bacteria was counted to test the antibacterial effect of the polyurethane foam (Liu et al., 2021).

Determination of ion content in water: A NexION300D inductively coupled plasma mass spectrometer (Perkin-Elmer, United States) was used to measure the K^+^, Na^+^, Ca^2+^, and Mg^2+^ content in seawater purified with the carbon-based polyurethane photothermal foam material.

## Results and Discussion

**FIGURE 1:**
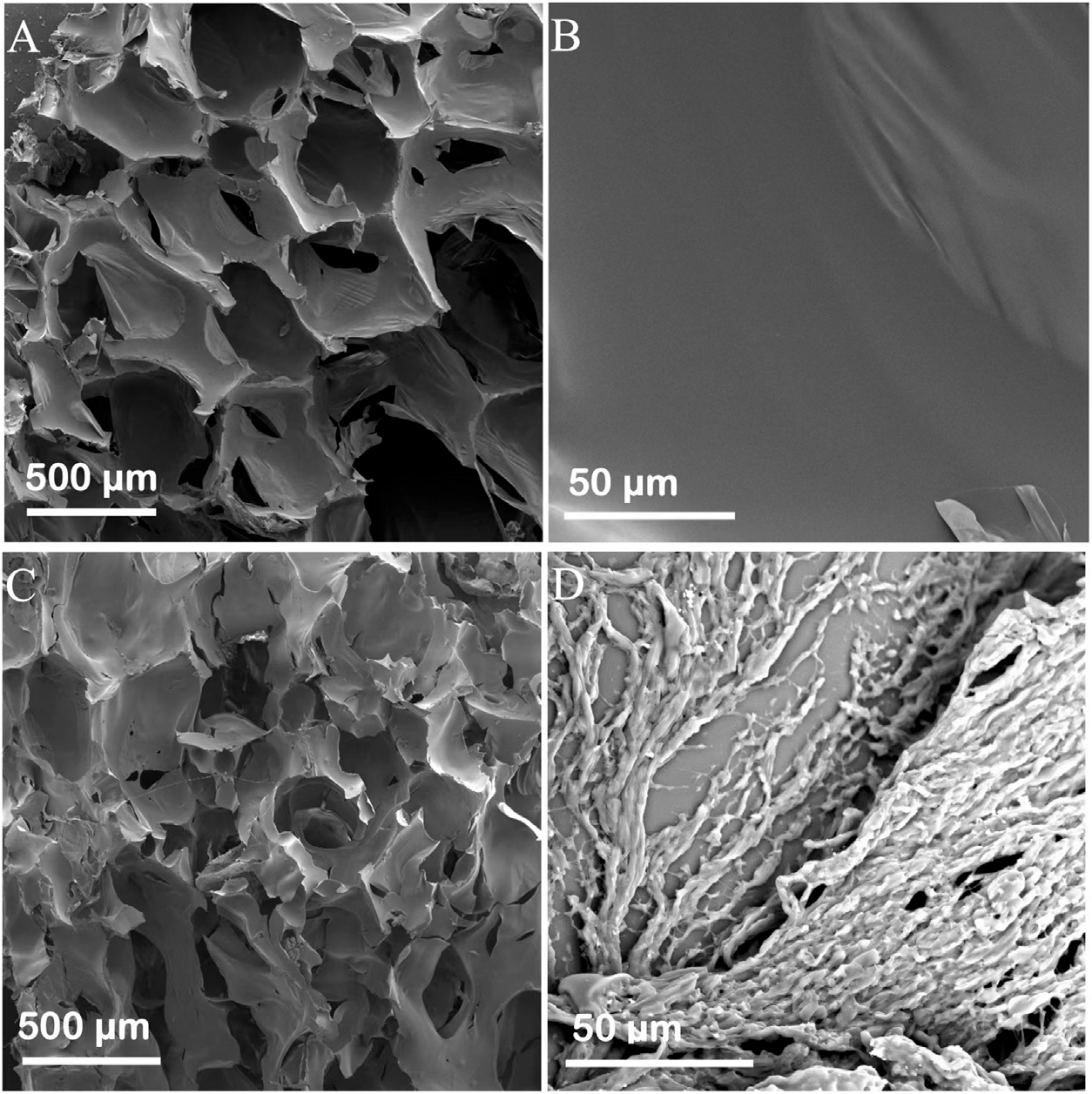
SEM images of **(A,B)** Ag and **(C,D)** Ag/PAM.

Figure 1 shows the scanning electron microscopy (SEM) images of the Ag and Ag/Polyacrylamide (PAM)-based foams. During the foaming process, large amounts of CO_2_ will be quickly generated. Thus, the obtained all foams will contain hierarchical and large-scale pore structures. In the Ag foam, the pores and wall were relatively smooth when compared with the Ag/PAM foam. while after *in situ* polymerization of PAM in the foam, the PAM can adhere on the pore surface, thus leading to the formation of fast water-transport channel and enabling an adequate water supply from the foam bottom to the surface. Compared with microporous and mesoporous structures, this macro-porous structure is more capable of improving the delivery efficiency of water, demonstrating certain advantages in the purification of high-concentration brine. Therefore, as the pore size increases, the pores are less likely to be blocked by salt crystals, resulting in a higher steam generation rate. Besides, the dispersion of various elements was further investigated by energy-dispersive X-ray spectroscopy (EDS, Supplementary Figure S2). EDS mapping revealed that in addition to carbon, nitrogen, and oxygen, abundant Ag was evenly dispersed in the Ag/ PAM-based foam, suggesting that photothermal performance of the foam could be uniform.

Fourier transform infrared spectroscopy (FTIR) was used to elucidate the formation of Ag- and Ag/PAM-based foam materials, as shown in Supplementary Figure S3. According to the figure, the peaks at 3,386 and 1,601 cm^−1^ are both attributed to absorption of -NH, while the positions at 1,354 cm^−1^, 1,223 cm^−1^, and 1,076 cm^−1^ are ascribed to the absorptions of –CH_3_, R–O–CH_3_, C–O–C, respectively. Moreover, the infrared bands of Ag/PAM at 3,700–3,500 cm^−1^ and 1850–1,430 cm^−1^ exhibited broadening and enhancement relative to those of the Ag material, indicating that the addition of PAM resulted in the formation of hydrogen bond in the Ag/PAM foam material.

Besides, the thermal stability and thermal conductivity of the foam were further investigated since photothermal materials are generally required to bear the heat and possess low thermal conductivity. As depicted in Supplementary Figure S4, the TG and derivative TG (DTG) curves of the Ag-based foams revealed that the weight loss gradually increased as the temperature for all of the samples. The PAM and PAM-containing syntactic foams exhibited an initial weight loss at approximately 90–180°C, which was caused by the evaporation of water molecules absorbed by PAM. The weight loss at 200–300°C was a result of the degradation of PAM side groups and the depolymerization of carbamate (Jeong et al., 2020), whereas the further degradation at 350–450°C was due to the degradation of the PAM main chain and the oxidation of the carbon residue. The thermal resistance of the carbon-based polyurethane foams is indicated by the thermal resistance temperature index Ts and the decomposition temperature Tc, as upheld by the International Standardization Organization (ISO), where Ts is calculated according to statistical methods using the formula T_s_ = 0.49 (T_d5_ + 0.6 (T_d30_ − T_d5_)) (Zhang J. et al., 2020), and Tc is the point where the line connecting the 20% and 50% weight loss points on the TG curve intersects with the extension line of the baseline. The results are presented in Supplementary Table S1. It can be observed from the figure and table that, after the addition of PAM, the foam exhibited reduced thermal stability.

**FIGURE 2:**
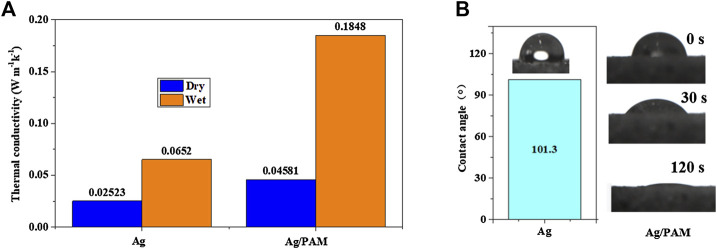
**(A)** Thermal conductivity and **(B)** water contact angles of Ag-based materials.

Thereafter, Figure 2A visualized the thermal conductivities of the silver-based foam materials. In the dry condition, the Ag material had a low thermal conductivity of 0.0252 W m^−1^ k^−1^, which was not significantly different from that of liquefied-chitin-based polyurethane foam (0.0267 W m^−1^ k^−1^). With the introduction of PAM, the thermal conductivity was mildly increased (lower than 0.05 W m^−1^ k^−1^). After water absorption in the wet state, the foam material, however, exhibited a sharply increased thermal conductivity. In particular, the Ag/PAM-based foam had a thermal conductivity of 0.1848 W m^−1^ k^−1^, which was ∼4 times higher than that of the dry-state material. However, the obtained value was still far lower than the thermal conductivity of water (0.599 W m^−1^ k^−1^). We hypothesized that the porous structures filled with large portions of air helped with low thermal conductivities for silver-based foams exhibited low thermal conductivities. By contrast, open pores provide a pathway for fluids to flow to the surface. Photothermal materials with low thermal conductivities help suppress local convection and concentrate the thermal energy (Ghasemi et al., 2014). Thus, the reduced heat conduction from the hot surface to the bulk water helped with the prevention of heat diffusion to the water body, finally minimizing heat losses from the bottom water (Dong et al., 2021).

Moreover, hydrophilicity is another key point for water transformation. Figure 2B; Supplementary Figure S5 show the water contact angles of the Ag, Ag/PAM foams and pure PAM, respectively. The Ag foam had a contact angle of 101.3°, indicating its poor hydrophilicity. While, the contact angle for PAM can rapidly reach 0° after 3 s. Therefore, after the *in-situ* formation of the PAM in the polyurethane foam, the hydrophilicity of foam was significantly improved. When water droplets were dropped onto the material surface, they were immediately immersed in the matrix within 2 min, suggesting that the hydrophilicity of foam was greatly enhanced by PAM. The porous structure and adhered PAM allow continuous and rapid transport of water molecules in the photothermal material, which facilitate the rapid escape of water vapor during evaporation (Liu et al., 2020; Zhou Q. et al., 2021).

As for photothermal materials, light absorption performance directly affects the final efficiency. Supplementary Figures S6A, S6B visualized the absorbance and reflectivity of the Ag and Ag/PAM-based foams. The figure reveals that the Ag and Ag/PAM foams had strong anti-reflection properties, with transmittance values less than 2% and reflection values less than 3% within a wide wavelength range of 250–2,500 nm. These properties indicate that the Ag and Ag/PAM foam materials have high light-absorption capabilities and can convert almost all of the incident sunlight energy into thermal energy, thereby achieving efficient water evaporation.

**FIGURE 3:**
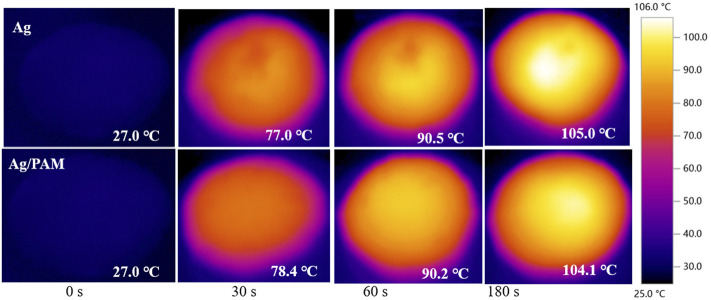
Infrared images of Ag and Ag/PAM materials under 1 sun illumination.

Based on above powerful light-absorption performance, the water-evaporation efficiency was then demonstrated. Supplementary Figure S7; Figure 3 show the material surface temperature maps and infrared images of the Ag and Ag/PAM foam materials under one-sun irradiation, respectively. According to the figure, the surfaces of the Ag and Ag/PAM foam materials with high solar absorption rates heated up rapidly under one-sun irradiation, with surface temperatures reaching 77.0 and 78.4°C from 27°C within 30 s, respectively. After continuous illumination for 180 s, the sample temperatures basically stabilized at ∼105.0 and ∼104.1°C, respectively, equivalent to temperature increases in the vicinities of 78.0 and 77.1°C. When solar irradiation was stopped, the silver-based foam material rapidly cooled to room temperature after 200 s. The rapid heating of the silver-based foams was mainly due to the strong solar absorption ability and low thermal conductivity, which supported the effective transport and fixture of solar energy and enabled rapid increases in the temperature.

In the photothermal water evaporation system designed herein, Ag acts as a photothermal absorber, whereas the Ag-based foam material converts absorbed solar energy into heat energy concentrated between the water interface and foam surface; this mechanism enables the foam temperature to increase rapidly, thus directly producing water steam.

**FIGURE 4:**
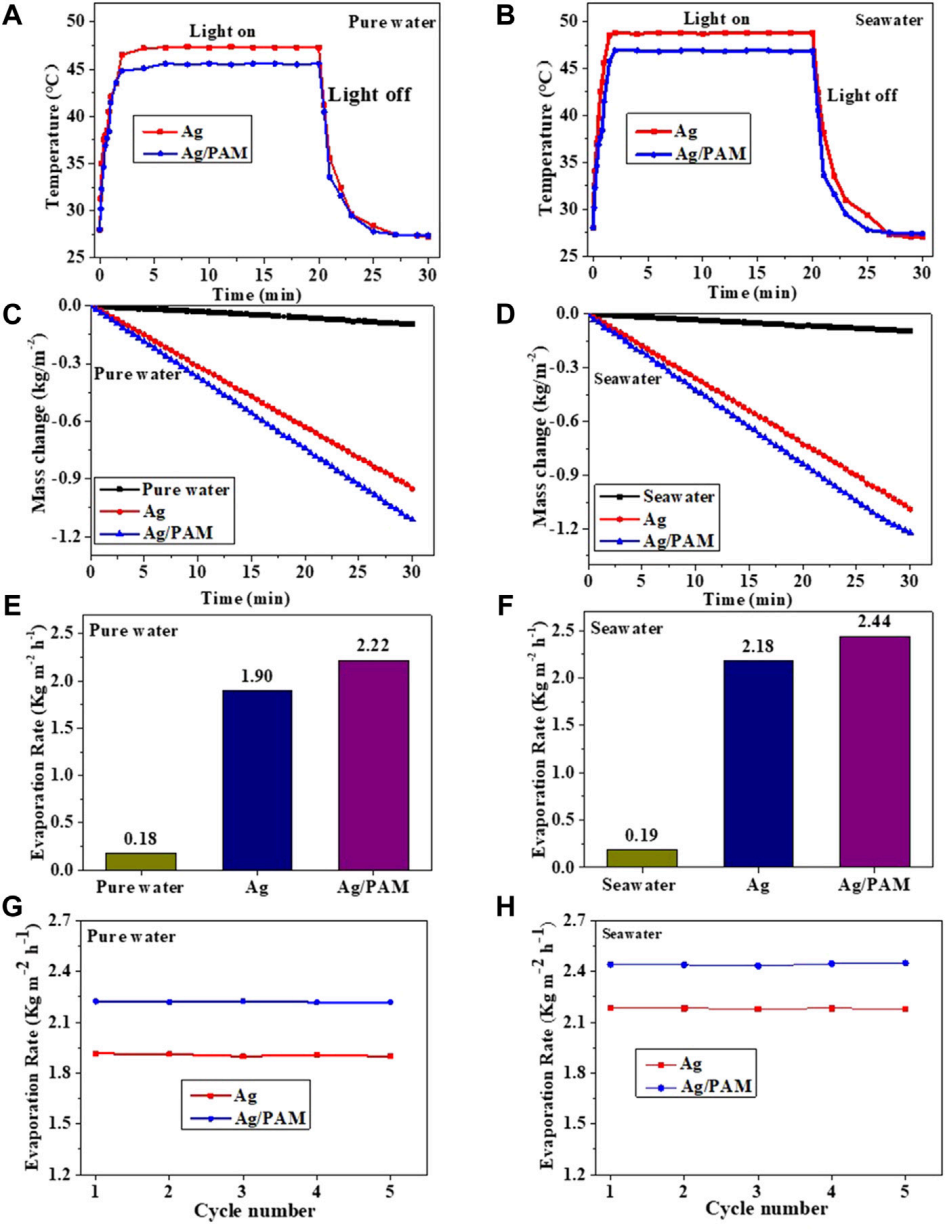
Ag and Ag/PAM materials under one sun irradiation: Time–temperature profiles of **(A)** Ag and **(B)** Ag/PAM; **(C)** Mass loss Ag and **(D)** Ag/PAM; Evaporation rate of **(E)** Ag and **(F)** Ag/PAM; Repeated evaporation rate of **(G)** Ag and **(H)** Ag/PAM.

The surface temperature change of the Ag-based foams involved two main stages (Figures 4A,B): heating and temperature stabilization. The first stage, with a spanning time of 0–3 min, is the heating stage, in which the absorptions of sunlight, solar conversion, and transformation of the adsorbed water processes happened in Ag-based foam, resulting in a continuous temperature increase. In this process, the heat loss was small because of the low thermal conductivity of the foam, resulting in a small temperature difference between the upper and lower material surfaces. The surface temperatures of the Ag and Ag/PAM foam materials increased rapidly from 27.0°C to 47.3, 45.6, 48.4, and 46.7°C, with increases ranging from 21.3 to 18.6°C, respectively. Therefore, there was almost no heat transfer from the foam to the water body owing to the low thermal conductivities and small heat losses of the foam.

The second stage, containing a spanning time of 3–20 min, is the surface temperature stabilization stage, in which heat conversion and heat transfer are balanced. In this stage, the system undergoes a large amount of water vapor evaporation. Since the foam conversion produces as much heat as that transferred to the water for water vapor production, both the surface and bottom temperatures of the Ag-based material were kept constant. During water evaporation, high water absorption ability of the polyacrylamide hydrogel allows the foam to quickly and continuously absorb a large amount of water and transport it to the surface through pores, leading to the acceleration of water evaporation and remove of heat. In both the dry and wet states, the polymer-based foam materials exhibited low thermal conductivities. Therefore, we briefly summarized the help of materials and structures in the foam, which is shown as follows:

1) The Ag-based foam materials can localize solar heat at the interface, reducing heat loss in the water body.
2) The polyurethane matrix with its low thermal conductivity can help suppress local heat convection and reduce heat loss.
3) The Ag-based foam material is in a semi-floating state in water. Therefore, the part near the air is semi-humid during solar steam generation. The semi-humid state and separation of the polyurethane matrix facilitate local heat generation and separation of interfacial water from the bulk water.
4) The open-pore structure in the Ag-based foam resulted in interconnected pores, providing a channel for water to flow to the interface, which effectively absorbs water to the material surface through capillary action. This superior capillary action guarantees the continuous replenishment of water supply to the hot interface, thus increasing photothermal vaporization.
5) The polymer-based foam materials contain polyacrylamide hydrogels, which support faster and sustained interfacial water transport that enhances the photothermal vaporization of water.

Moreover, Figures 4C,D show the relationship between water losses and irradiation times of the polymer-based foam materials in pure water and seawater under one-sun irradiation by an electronic analytical balance. The water evaporation rates (Figures 4E,F) were calculated based on the slope of the curve. After 1 h of stable evaporation, the Ag and Ag/PAM materials were determined to have evaporation rates of 1.90 kg m^−2^ h^−1^ and 2.22 kg m^−2^ h^−1^, respectively, in pure water, while the obtained results in seawater were 2.18 kg m^−2^ h^−1^ and 2.44 kg m^−2^ h^−1^, respectively (Cui et al., 2018). In other words, under one-sun irradiation, each material exhibited a slightly higher evaporation rate in seawater than in pure water. The solar evaporation stabilities of the polymer-based foams were investigated based on five repetitions of the water evaporation experiment under one-sun irradiation (Figures 4G,H). The evaporation rate has a cyclic variation below 2%, suggesting that the foam material has high solar evaporation performance and good evaporation stability (Guo et al., 2020).

For deeply understand the evaporation mechanism, the evaporation enthalpy of absorbed water was thereafter studied. Solar steam conversion efficiency can be calculated in several ways. In this study, two different methods were used to calculate solar steam conversion efficiency. The first method is to calculate according to Eq. 1 in the paper by Wang (Zhang et al., 2015), where Qs is the incident light power, and Qe is the water evaporation power, which is estimated based on Eq. 2, where m is the evaporated water mass, t is time, v is the water evaporation rate, and He is the water evaporation heat (∼2,260 kJ kg^−1^). Based on the aforementioned formulae, the solar energy conversion efficiencies of the carbon-based materials in pure water and seawater under one-sun irradiation were calculated, and the results are shown in Table 1.

η=Qe/Qs (1)

$$Q_{e}=\frac{dm\times H_{e}}{dt}=\nu \times H_{e}$$ (2)

**TABLE 1:** The energy conversion efficiency of Ag and Ag/PAM materials under one Sun irradiation with pure water and seawater.

	Ag	Ag /PAM
η_water_ (%)	119.28	139.37
η_seawater_ (%)	136.86	153.18

It has also been reported that if the solar-absorbing material loses heat to the environment, the final calculations will disestablish the theoretical energy equilibrium. By contrast, if the hot-water region temperature near the evaporation surface is considered, the results will be more reasonable and consistent with experimental observations. Hence, this experiment also adopts another method to determine the solar energy conversion efficiency based on the proportion of solar energy consumed in steam generation, and the following formula is used for calculation (Zhao et al., 2018; Wu et al., 2019; Zeng et al., 2019; Zhang Y. et al., 2020; Zhou S. et al., 2021):

$$\eta =\frac{\dot{m}\left(H_{lv}+Q\right)}{C_{opt}q_{solar}}$$ (3)

$$H_{lv}=1.91846\times 10^{6}{\left[\frac{T_{i}}{T_{i}-33.91}\right]}^{2}$$ (4)

Q=Cp(Ti−Ts) (5)

where Copt q_solar_ is the total solar energy input (Copt is the light concentration, which is 1 kW m^−2^, and q_solar_ is the solar radiation on the surface, 1 Kw m^−2^), and $\dot{m}$ is the water evaporation rate (kg m^−2^ h^−1^). In the calculation for $\dot{m}$ , it is necessary to subtract natural evaporation under dark conditions from the experimentally derived evapotranspiration flux under illumination, such that the evaporation rate used in the calculation will be based on only the illumination. Meanwhile, (Hlv + Q) is the total enthalpy required for liquid water to change into steam, and includes the sensible heat of water (Q) and the latent heat of the liquid–gas phase change (Hlv). Moreover, Hlv is the latent heat required for water evaporation (kJ kg^−1^) and depends on the water/air interface temperature required for vaporization; Ti is the temperature of the water/air interface (K); Q is the heat required to raise the water temperature, i.e., the sensible heat of water, which depends mainly on the specific heat of water and the temperature difference between the solar absorber and water body; Cp is the specific heat capacity of water (4.186 kJ kg^−1^ K^−1^); Ts is the initial water temperature, which was 27°C in this experiment; and Ti is the experimentally measured temperature of the water. Based on the aforementioned formula, it is hypothesized that the energy efficiency of the solar steam is 100%. If the corresponding evaporation rate is 1.43 kg m^−2^ h^−1^, then the actual evaporation rates of the photothermal materials under one-sun irradiation were approximately 133–139% of the theoretical limit. In this experiment, based on the surface temperatures of the carbon-based materials, specific heat of water, latent and sensible heats of water evaporation, and functional relationship of the energy efficiency under one-sun radiation, the theoretical value was estimated based on an energy efficiency of 100%, to enable the calculation of the water evaporation efficiency; the results are shown in Tables 2, 3. The tables reveal that both the freshwater and seawater evaporation efficiencies of the Ag and Ag/PAM foam materials exceeded 100%. In this experiment, a polyurethane foam with low thermal conductivity and a hydrophilic gel applied into the pores were used as porous support to prevent direct contact between the photothermal material and bulk water, thus reducing the loss of generated heat and significantly improving the water evaporation efficiency of the polymer-based foam material.

**TABLE 2:** The surface temperature, latent heat, sensible heat, evaporation enthalpy, and energy conversion efficiency of Ag and Ag/PAM materials under one Sun irradiation with pure water.

	Ag	Ag /PAM
Surface temperature (°C)	49.0	46.8
Latent heat (kJ kg^−1^)	2,396.41	2,400.29
Sensible heat (kJ kg^−1^)	92.40	83.16
Evaporation enthalpy (kJ kg^−1^)	2,488.81	2,483.45
Energy conversion efficiency η (%)	122.36	144.18

**TABLE 3:** The surface temperature, latent heat, sensible heat, evaporation enthalpy, and energy conversion efficiency of Ag and Ag/PAM materials under one Sun irradiation with seawater.

	Ag	Ag /PAM
Surface temperature (°C)	47.3	45.6
Latent heat (kJ kg^−1^)	2,399.40	2,402.43
Sensible heat (kJ kg^−1^)	85.26	78.12
Evaporation enthalpy (kJ kg^−1^)	2,484.66	2,480.55
Energy conversion efficiency η (%)	141.49	159.17

In the experiment, DSC was used to verify the evaporation enthalpy of water, and the results are shown in Supplementary Figure S8. The figure shows that the heat flow signal varied with temperature. When pure water was used as the control sample, the maximum value was reached, in which a peak was observed in the thermal signal (the signal suddenly dropped), indicating that water evaporation was soon to be completed. By contrast, the polymer-based foam materials exhibited wider and gradually decayed peaks, demonstrating that evaporation behaviors of the foam were different from that of pure water. As shown in Table 4, the water evaporation enthalpy obtained in this experiment was 2448 J g^−1^, which was close to the theoretical value of 2448 J g^−1^, illustrating the measurement accuracy of this experiment. The polymer-based foam material exhibited significantly lower evaporation enthalpies than that of pure water. In particular, after the *in-situ* generation of hydrogel in the polyurethane foam, the evaporation enthalpy became even lower. In this experiment, DSC measurements supported only a qualitative evaluation of the reduction in evaporation enthalpy due to the polymer-based foam material. Therefore, DSC revealed the complete dehydration process of the foam, including the energy required to evaporate the three types of water (bound, intermediate, and free water). Because of the reduced water evaporation enthalpy, intermediate water had a significantly higher evaporation rate than that of free water in the silver-based foam materials. In addition to the amino, cyano, and –OH groups from the polyurethane and chitin liquefaction products, which formed strong hydrogen bonds with water, the PAM also formed strong hydrogen bonds with water molecules. Thus, more water molecules form weak hydrogen bonds with surrounding water molecules. These surrounding water molecules are intermediate water molecules characterized by weak interactions with the polymer chain and adjacent water molecules. Because the water clusters formed by hydrogen bonds and the hydrogel molecular network are easily evaporated under solar irradiation, the water evaporation enthalpy is effectively reduced. For instance, water molecules approaching the chitin liquefaction product and polyurethane chains can form strong hydrogen bonds, which weaken the hydrogen bonds of adjacent water molecules, thus facilitating the evaporation of these molecules into the air. However, for inorganic materials with large porous channels, water usually has a low level of evaporation because of the accumulation of a large amount of water in the channels, which can be regarded as dispersed bulk water that hinders water evaporation (Zhao et al., 2018; Zhou et al., 2018; Guo et al., 2019; Zhou et al., 2019). In this experiment, the addition of hydrogel increased the proportion of intermediate water and further reduced the water evaporation enthalpy. Simultaneously, the formation of micropores broke water clusters, which accelerated the evaporation rate of the polymer-based foam material and increased the evaporation efficiency.

**TABLE 4:** Evaporation enthalpy results from DSC measurement of Ag and Ag/PAM.

Sample	Water	Ag	Ag/PAM
Enthalpy (J g^−1^)	2,448	1,191	1,139

Additionally, owing to the introduction of nano-Ag, the obtained foam displayed excellent antibacterial properties, and the results are shown in Supplementary Figure S9. As shown in Supplementary Figure S9A, the Ag displayed excellent anti-bacterial performance since no bacterial existed. As expected (Supplementary Figure S9B), the Ag-based foam also exhibited significant antibacterial performance since silver nanoparticles can adhere to the surfaces of the cell walls and cell membranes of bacteria, penetrate into the cells, destroy internal cell structures, and induce cytotoxicity. On the other hand, we believe that the chitin, including small and large-molecular-weight molecules, in the chitin liquefaction product would also act on the bacterial surface and affects its metabolism. The excellent antibacterial effect of the polymer-based foam materials can provide resistance to biological contamination. Furthermore, without bacterial attachment blocking the pores of the foam, it is possible to maintain a long-lasting and efficient water evaporation performance in the material (Li et al., 2019).

With seawater from the South China Sea as the water source, the concentrations of Na^+^, Mg^2+^, K^+^, and Ca^2+^ were investigated before and after seawater desalination, and the results are shown in Supplementary Figure S10. The results indicate that the concentrations of Na^+^, Mg^2+^, K^+^, and Ca^2+^ in the water were reduced by desalination from 218 mg ml^−1^ to 201 mg ml^−1^ to below 1, respectively, which are far lower than the salinity standard for potable desalinated water stipulated by the World Health Organization (Wu et al., 2019; Wang et al., 2021).

## Conclusion

In conclusion, based on five principles, the nano Ag-based photothermal foam with excellent light absorption and low thermal conductivity was prepared in this work. Under one-sun irradiation for 3 min, the temperatures on the foam can increase to as high as 105 and 49°C from room temperature in the air and water conditions, respectively. In comparison, the evaporation masses of the Ag and Ag/PAM based materials can reach 2.18 and 2.44 kg m^−2^ h^−1^, respectively, in seawater under one-sun irradiation. The water evaporation efficiencies, reaching 136.9% and 153.2%, respectively, are also superior to those of most metal-based photothermal materials reported in the literature.

## Data Availability

The original contributions presented in the study are included in the article/Supplementary Material, further inquiries can be directed to the corresponding author.
