# Supplementary material for: Rational Design of Photothermal and Anti-Bacterial Foam With Macroporous Structure for Efficient Desalination of Water
Source: Front Chem. 2022 May 11;10:912489. doi: 10.3389/fchem.2022.912489 (PMC9130493; doi:10.3389/fchem.2022.912489)
Supplement: Supplementary file 1 [file DataSheet1.docx]

Supplementary Material

**Rational Design of Photothermal and Anti-Bacterial Foam with Macroporous Structure for Efficient Desalination of Water**

**Zhifen Wang^1^, Jin Niu^1^, Juanxia Wang^1^, Yucang Zhang^2^*, Guoqiang Wu^1^, Xiaoyun Liu^1^, Qun Liu^2^**


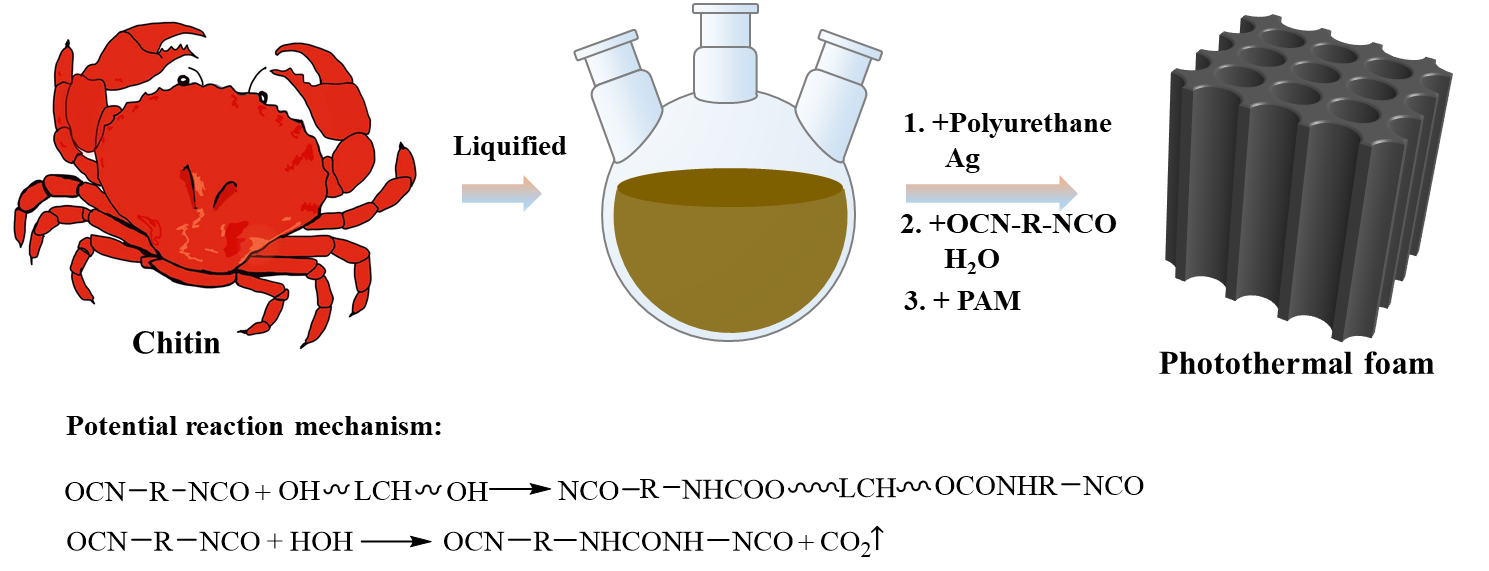


Figure S1 Preparation process of the photothermal foam and the potential reaction mechanism during the synthesis.


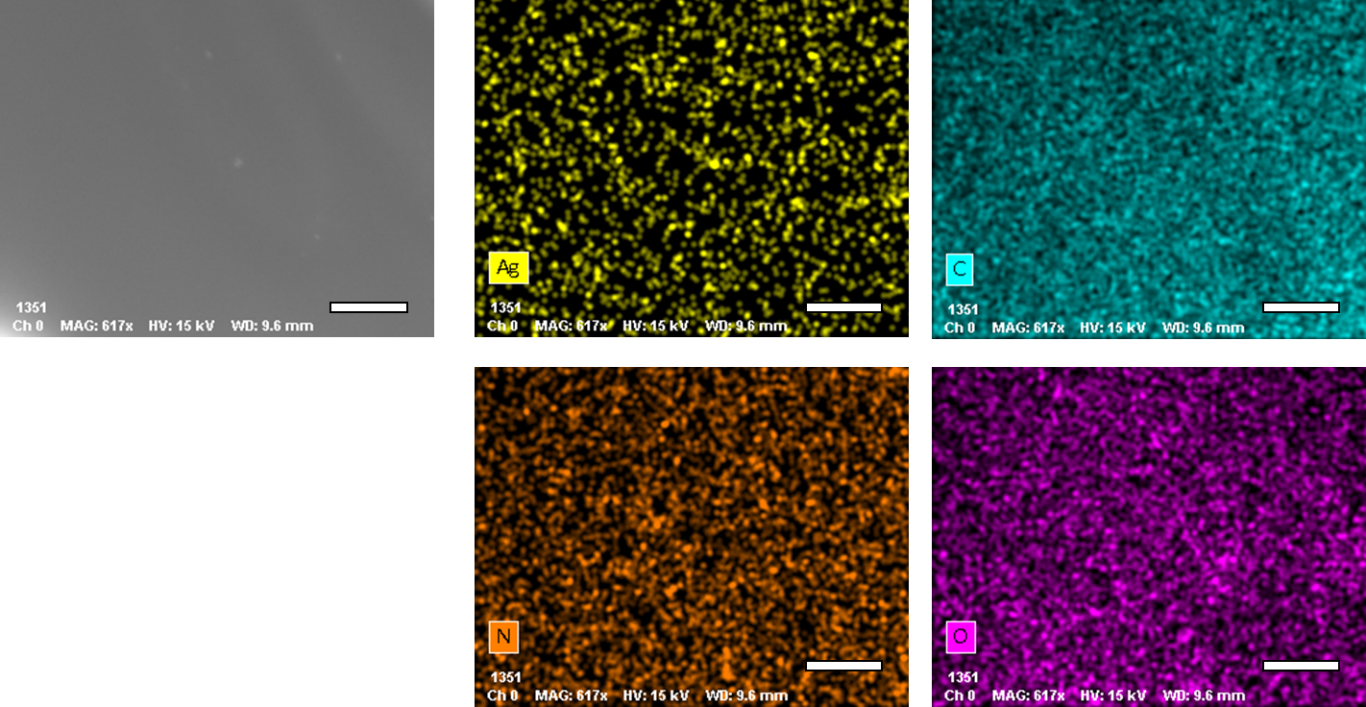


Figure S2 SEM image and EDS element maps of Ag/ PAM-based foam, and the scar bar is 20μm.

Figure S3 FTIR spectra of PAM and Ag-based materials


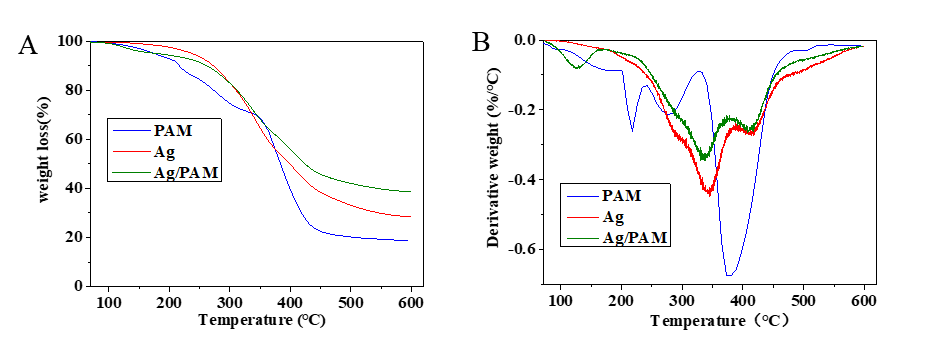


Figure S4 (A) TG and (B) DTG curves of Ag-based materials.


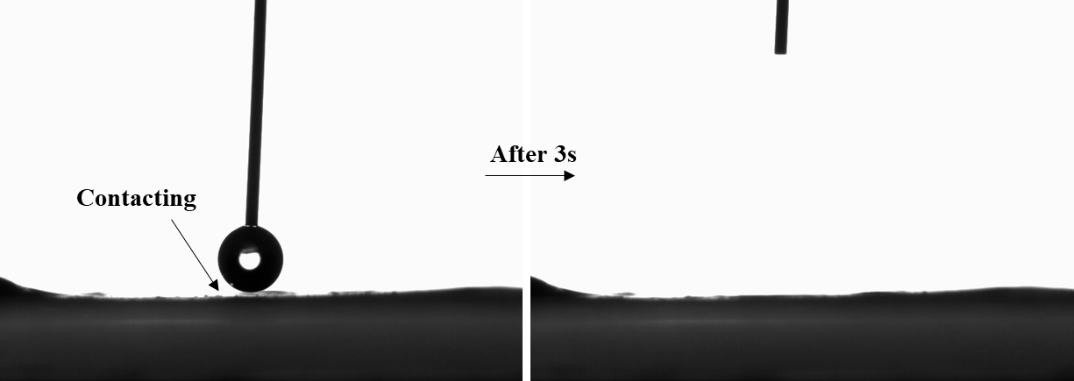


Figure S5 Water contact angles of pure PAM.


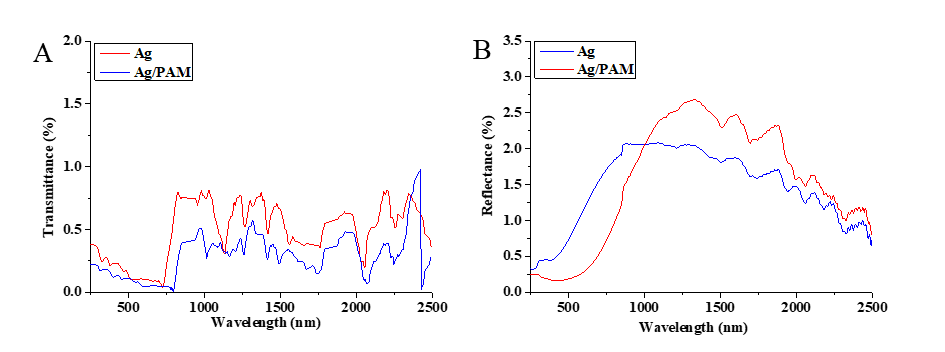


Figure S6 UV–vis–NIR spectra of Ag and Ag/PAM materials in the wavelength range of 250-2500 nm. (A) transmittance; (B) reflectance.

Figure S7 Temperature response profiles of Ag and Ag/PAM materials when 1 sun irradiation turns on and off

Figure S8 Thermograms of pure water, Ag, and Ag/PAM materials.


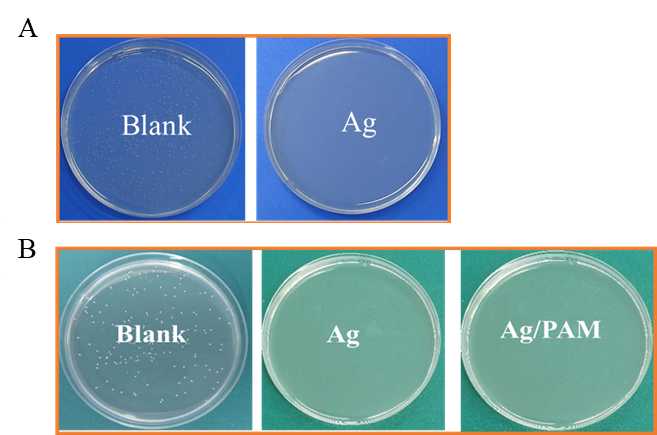


Figure S9 (A) Antibacterial tests of pure nano silver and (B) antibacterial tests of Ag and Ag/PAM foams.

Figure S10 Ion concentrations (Na^+^、Mg^2+^、K^+^、Ca^2+^）in seawater before and after desalination by Ag and Ag/PAM.

Table S1 TGA data of carbon-based materials

| Samples | Td5 (℃) | Td30 (℃) | Ts (℃) | Tc (℃) | Char (%) |
| --- | --- | --- | --- | --- | --- |
| PAM | 174.2 | 341.7 | 134.7 | 201.4 | 18.6 |
| Ag | 236.5 | 337.5 | 145.6 | 251.8 | 25.1 |
| Ag /PAM | 180.0 | 344.1 | 136.4 | 236.5 | 32.5 |
